# Hydrogels Based on Natural Polymers Loaded with Bentonite and/or Halloysite: Composition Impact on Spectroscopic, Thermal, and Swelling Properties

**DOI:** 10.3390/molecules29010131

**Published:** 2023-12-25

**Authors:** Rodrigo César Sabadini, Mariana Fernandes, Verónica de Zea Bermudez, Agnieszka Pawlicka, Maria Manuela Silva

**Affiliations:** 1 Centro de Química e Departamento de Química, Universidade do Minho, Campus de Gualtar, 4710-057 Braga, Portugal; rodoxxx@gmail.com; 2 Instituto de Química de São Carlos, Universidade de São Paulo, Av. Trabalhador Sãocarlense 400, São Carlos 13566-590, SP, Brazil; 3 Department of Chemistry and CQ-VR, University of Trás-os-Montes e Alto Douro, 5000-801 Vila Real, Portugal; mspf@utad.pt (M.F.); vbermude@utad.pt (V.d.Z.B.)

**Keywords:** hydrogels, nanocomposites, gellan gum, starch, bentonite, halloysite

## Abstract

Hydrogels are characterized by their property of absorbing and releasing a high content of water and water-based liquids; thus, they can be applied in agriculture as controlled-release water and fertilizer products. The focus of this research was efficient and low-cost natural polymer-based hydrogels obtained by crosslinking gellan gum (GGLA) and starch (ST) with acetic acid (CA) and loading them with either bentonite (BET) and/or halloysite (HAL). The hydrogels were obtained by mixing 100, 75, 50, 25, and 0 wt.% of GGLA with 0, 25, 50, 75, and 100 wt.% ST water solutions. To obtain the networks, they were crosslinked with 10, 5, and 2 wt.% of CA and loaded with 2, 5, and 10 wt.% of BET and/or HAL. The samples were analyzed by infrared spectroscopy (FTIR), differential scanning calorimetry (DSC), their swelling in water, and the state of bound water properties. The results of these analyses point to the formation of a polymeric network with a decomposition temperature of >250 °C, and tailorable swelling properties that vary between 3 and 77, depending on the hydrogel composition. In summary, GGLA-ST-BET/HAL hydrogels are a good option for eco-friendly agriculture materials.

## 1. Introduction

Agroecology studies are related to the four properties of agroecosystems: productivity, stability, sustainability, and equitability, and these properties can be studied at any spatial scale, from small to continental cultures [1]. To improve and optimize agricultural production systems, studies have shown that the use of hydrogels can manage water better in crops [2,3]. Natural polymer-based hydrogels have also been widely investigated for smart drug delivery platforms [4] because of their high swelling properties and pH responsiveness [5,6].

Hydrogels are defined as polymer networks, which can reversibly absorb and release water solutions. Because they have hydrophilic groups in their network, they can swell when put in an aqueous solution [7,8]. Hydrogels can be classified into three classes depending on the nature of the polymer chains crosslinking, which can be chemical, physical, and a combination of both with different proportions. The crosslinks made through chemical reactions lead to covalent bond formation between the macromolecular chains. Usually, this is achieved through some crosslinking agent and, as a result, a polymer network is formed [9].

Clays and clay minerals are widely used by farmers and industry because of their adsorption, swelling, rheological, colloidal, and plasticity properties, among others. Therefore, their uses and applications extend from ceramics, oil, and paper, to metallurgical industries [10]. Additionally, clay minerals have attracted attention due to certain properties such as their layered structure with a nanoscale dimension, a layer thickness ranging from 0.7 to 1.0 nm, the anisotropy of their layers or particles, their easily modifiable outer and inner surface, which can be achieved by grafting, adsorption, or ion exchange, and their plasticity and hardening achieved by drying, which is possible in most clay minerals [11].

Bentonite (BET) is an absorbent aluminum phyllosilicate clay comprised mostly of montmorillonite (MMT). It is also the commercial name for MMT and has been used for polymer–clay nanocomposite synthesis. Depending on the clay, they can have different chemical compositions and form different structures, which can impact the polymer–clay interaction. The montmorillonite “theoretical” chemical formula is (Al_3.33_Mg_0.67_)Si_8_O_20_(OH)_4_ M^+^_0.67_, where M^+^ is a monovalent cation. This clay unit cell has a negative electric charge because of the isomorphic substitution of Al^3+^ for Mg^2+^, and the M^+^ cation is exchangeable and balances the negative charge [12]. Halloysite (HAL) is an aluminum silicate clay mineral [13] in the form of nanotubes and has a chemical formula Al_2_Si_2_O_5_(OH)_4_·nH_2_O [14]. Its small particle size and the ability to mix or modify them with natural polymers such as chitosan [13] or those containing starch [14] make them a material of great application potential as well as a green alternative to the current synthetic polymer-based hydrogels [15]. 

The use of macromolecules for the preparation of hydrogels usually requires the product to be non-toxic, biocompatible, and biodegradable. Natural polymers display these characteristics, so they can be used for such purposes. As some examples, we can cite alginate, chitosan, collagen, cellulose, gellan gum, and many others [16]. However, the natural polymers’ different characteristics are reflected in their hydrogels. To improve these natural polymer-based hydrogel properties, the incorporation of inorganic fillers into a polymer matrix results in optimized materials with improved physical properties, transposing the limitations of the pure polymer [17,18]. The incorporation of inorganic fillers with nanometric dimensions—such as lamellar silicates—has been widely used by the scientific and technological community [19]. Nanocomposite hydrogels have been prepared for several purposes including controlled release [20], scaffolds [21,22], water purification [23,24], etc. Dhibar at al. [25] have shown that the addition of small quantities of 3 to 5% of nanoscale clay to the polymer matrix improves its thermal and mechanical properties when compared to 30 to 50% of microclay addition. 

For the preparation of nanocomposite hydrogels, it is possible to crosslink polymers with suitable crosslinking agents and add the clay, or use modified clays, which usually have an opposite charge to the polymer. Alternatively, bentonite, montmorillonite, sepiolite [26], or halloysite [13,14] are good candidates for such a purpose. Therefore, the modified clay itself may serve as a crosslinker [27,28]. Many crosslinker agents are toxic and/or expensive and are not always suitable for such purposes. On the other hand, citric acid (CA) is a cheap chemical compound and can crosslink certain polymer chains [29,30,31]. It is used to covalently crosslink the carboxylic with hydroxyl groups of polysaccharides through an esterification reaction [32].

In this work, we present the results of hydrogels prepared with natural polymers, namely, low acyl gellan gum and starch that were crosslinked with citric acid and loaded with either bentonite and/or halloysite. The obtained samples were characterized by FTIR spectroscopy, DSC thermal analysis, and water swelling. The samples’ state of bound water was also analyzed.

## 2. Results and Discussion

### 2.1. GGLA–ST, GGLA–ST–BET, and GGLA–ST–HAL Hydrogels

GGLA is a product of the alkaline treatment of native gellan gum, in which most of the acyl groups are removed. This results in a brittle, firm, and optically clear gel. Hydrogels based on GGLA are brittle and transparent and present with a lower water absorption if compared with hydrogels prepared with native gellan gum [9,33].

Bentonite (BET) is a natural hydrophilic clay with a lamellar structure constituting mostly of montmorillonite. It has been used in some polymer–clay nanocomposites due to its capacity to absorb a large amount of water between its unit layers; thus, it can improve the water retention/release proprieties of soils [34,35].

Halloysite (HAL) is an aluminosilicate clay mineral with formula Al_2_Si_2_O_5_(OH)_4_ and consists mainly of Al (~21%) and Si (~22%). Typically, it is obtained through the hydrothermal deformation of sheets into tubes that have an external diameter of 50 to 70 nm. These nanotubes are a viable and inexpensive material for the absorption and release of controlled substances [36].

### 2.2. Fourier-Transform Infrared Spectroscopy (FT-IR)

**Figure 1:**
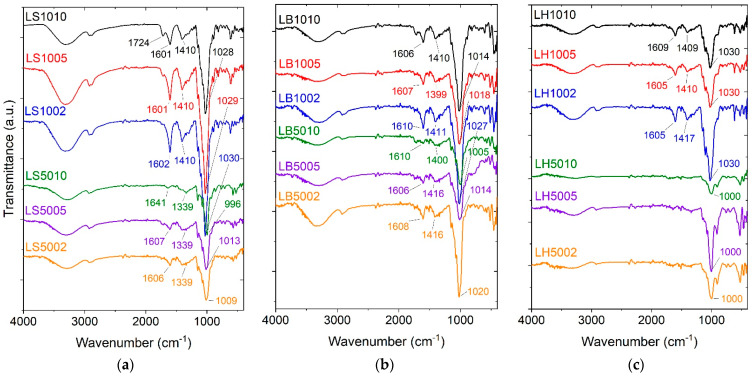
FTIR spectra of hydrogels LS10ZZ and LS50ZZ (**a**), LB10ZZ and LB50ZZ (**b**), LH10ZZ and LH50ZZ (**c**), crosslinked with ZZ = 10, 5, and 2 wt.% of CA.

Figure 1a shows the FTIR spectra for samples LS10ZZ and LS50ZZ, Figure 1b for the bentonite-loaded samples (LB10ZZ and LB50ZZ), and Figure 1c for the halloysite-loaded samples (LH10ZZ and LH50ZZ). All these samples were crosslinked with three concentrations (ZZ = 10, 5, and 2 wt.%) of CA.

Pure gellan gum has characteristic peaks at 3425 cm^−1^ as a result of the H-bonded O–H stretch vibration of its hydroxyl groups. At 2931 cm^−1^, there are C–H stretch vibrations of CH_2_ groups. Asymmetric carboxylate anion stretching is at 1610 cm^−1^. At 1417 cm^−1^, there is symmetric carboxylate anion stretching, and, at 1033 cm^−1^, C–O stretching [33]. The GGLA and ST hydroxyl groups’ vibration is seen in all the spectra as a broad band at ~3400 cm^−1^; C-O stretching is seen at 1028 cm^−1^ (Figure 1) [14]. This band shifts to 1013–1009 cm^−1^ for the samples with 50% of ST and 2 and 5 wt.% of CA (Figure 1a). However, as the crosslinking of the GGLA with CA occurs through esterification, a peak at ~1000 cm^−1^ can also be due to the CO-C-CO stretch [37]. The peaks at ~1600 cm^−1^ could be a result of the GGLA unreacted COOH or the C=O stretching of CA, and they probably coincide with glycosidic bonds. The peaks at ~1400 cm^−1^ very likely overlay the peak of the C–H stretching in the polymer film [38,39]. 

Typical CA absorption bands are seen at 1690 and 1743 cm^−1^ and are assigned to free and H-bounded COOH groups, respectively [40]. The indication of the reaction between the COOH groups of CA and the NH_2_ groups of GGLA is seen by the vanishing of the peak at 1743 cm^−1^ in almost all the samples except for LS1010, which displays a peak at 1724 cm^−1^ (Figure 1a). 

For the samples without BET or HAL, i.e., LS1010, LS1005, and LS1002, there are corresponding bands at 1030, 1029, and 1030 cm^−1^ (Figure 1a). These bands are from the stretching vibrations of C-OH, CO-C-CO [37], and the other acetate ligands of GGLA [41]. After the addition of bentonite, these bands shifted to 1014, 1018, and 1027 cm^−1^ for LB1010, LB1005, and LB1002, respectively (Figure 1b). Similar shifts of the bands at 996, 1013, and 1009 cm^−1^ are observed for LS5010, LS5005, and LS5002, respectively (Figure 1a). These bands are the result of the stretching vibrations of alcohol C-OH and CO-C-CO [37] and also other acetate ligands of GGLA [41]. They have shifted to 1005, 1014, and 1027 cm^−1^ for LB5010, LB5005, and LB5002, respectively (Figure 1b).

The intense peak at ~1000 cm^−1^ in the LH samples (Figure 1c), similarly to the samples without BET or HAL (LSYYZZ) and with bentonite (LBYYZZ), is associated with the CO-C-CO stretch [39] because of the crosslinking esterification reaction. This peak can also overlap with the Si-O-Si vibrations of halloysite [13]. Small peaks at ~1600 cm^−1^ could be due to the reaction between the carboxylic groups of citric acid and the hydroxylic groups of ST and GGLA, and the peaks at ~1400 cm^−1^ can be result of the polymer C–H stretching superposition peak [38,39].

### 2.3. Differential Scanning Calorimetry (DSC)

**Figure 2:**
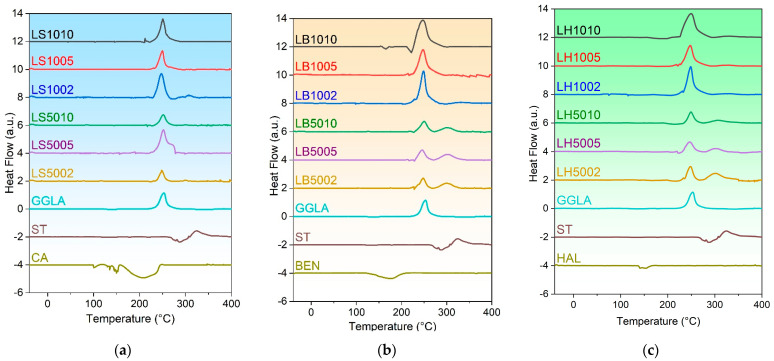
DSC curves for samples LS10ZZ, LS50ZZ, GGLA, ST, and CA (**a**), LB10ZZ, LB50ZZ, GGLA, ST, and BET (**b**), and LH10ZZ, LH50ZZ, GGLA, ST, and HAL (**c**); hydrogels were crosslinked with ZZ = 10, 5, and 2 wt.% of CA.

Figure 2a shows the DSC curves for the GGLA-ST hydrogels crosslinked with 10, 5, and 5 wt.% of CA, and Figure 2b shows the DSC results for the GGLA-ST-BET samples crosslinked with different percentages of CA. The DSC curves for GGLA-ST-HAL with a different percentage of CA crosslinker are displayed in Figure 2c.

Thermograms in Figure 2a–c show the degradation peaks at about 250 °C, typical for polysaccharide behavior [42,43]. The thermal pretreatment of the samples eliminated the typical peaks for the polysaccharides´ water loss. We did not observe the glass transition (Tg) of the prepared hydrogels due to the temperature range used for this analysis. Another reason for this could be because of its strong intra- and intermolecular hydrogen bonding and rigid dry structure [44].

### 2.4. Swelling Degree

One of the key features of a hydrogel is its swelling when in contact with water or other compatible solvents. The hydrophilic chains in the hydrogel absorb water due to hydration forces and are counterbalanced by the forces of the crosslinked chain. When these forces are equal, the equilibrium is reached and the maximum swelling is achieved [9,33].

**Figure 3:**
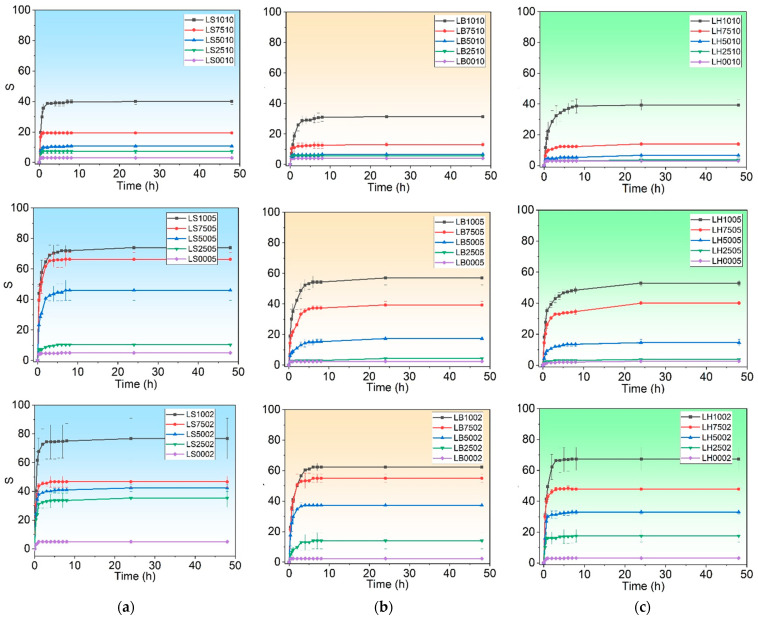
Swelling degree (S) for hydrogels LS10ZZ and LS50ZZ (**a**), LB10ZZ and LB50ZZ (**b**), LH10ZZ and LH50ZZ (**c**), crosslinked with ZZ = 10, 5, and 2 wt.% of CA.

The swelling degree (S) for the hydrogels with 100, 75, 50, and 25 wt.% ratios of (i) GGLA to ST and 10, 5, or 2 wt.% of CA added as a crosslinking agent were calculated using Equation (1), and are presented in Figure 3a; (ii) GGLA-ST-BET and 10, 5, and 2 wt.% of CA are in Figure 3b; and (iii) GGLA-ST-HAL and 10, 5, and 2 wt.% of CA are displayed in Figure 3c.

Figure 3 shows that the maximum absorption occurs after 24 h of all the samples’ immersion in water, independent of whether they were loaded or not with BET and/or HAL. The samples crosslinked with a lower amount of citric acid presented higher swelling values because of the fewer crosslinking points [9,33]. The maximum absorption occurred in the samples with 2 wt.% of CA. The same behavior has been observed in other hydrogels described in the literature [45,46]. Besides that, the higher the percentage of gellan gum, the higher the maximum absorption of the hydrogel, where the hydrogels composed of 100% gellan gum registered an S of 40, 74, and 77 for samples with 10, 5 and 2% CA (Figure 3a). The samples with less crosslinks, which means less CA, displayed higher S values, that are comparable with methacrylated gellan gum hydrogels [34]. On the other hand, the 100% starch hydrogels presented the lowest water absorption, with S values between 3 and 5 (Figure 3a), which is also consistent with results published in the literature [31]. The samples with 50%GGLA−50%ST presented S values of approximately 40 for 5 and 2% CA. The samples of copolymerized acrylamide with acrylic acid, β-cyclodextrin, and 1 and 2 wt.% of starch have shown S ~52 after 24 h [14].

The maximum S values for the 100% gellan gum−10% BET were 31, 57, and 62 for the samples with 10, 5, and 2% added CA, respectively (Figure 3b). The addition of BET decreased the maximum absorption, if compared to the samples without BET and/or HAL. Despite being hygroscopic, BET may influence the polymeric chain elasticity and decrease the overall swelling [46]. As expected, the 100% starch hydrogels also presented the lowest swelling, but the samples prepared with 50–50% gellan gum–starch presented good S values of 17 and 37 for the samples with 5 and 2% added CA, respectively.

Maximum swellings of 39, 53, and 67 were obtained for the samples composed of 100% gellan gum−10% HAL crosslinked with 10, 5, and 2% of citric acid (Figure 3c). It should also be mentioned that the addition of halloysite decreases the maximum absorption when compared to the samples without BET and/or HAL, and the same behavior has already been observed in nanocomposite–bentonite hydrogels. This is probably due to the BET and/or HAL’s influence on the polymeric chain elasticity and results in an overall swelling decrease [46]. As expected, the 100% starch-based hydrogels also presented the lowest swelling, but the samples prepared with 50–50% gellan gum–starch presented good swelling values of 14 and 36 for the samples with 5 and 2% added citric acid, respectively. This was also very similar to the nanocomposite bentonite hydrogels. 

The samples presented in this work had 4–5 times lower swelling values when compared to the samples containing high acyl gellan gum, starch, and BET and/or HAL [47]. This was also observed in other hydrogel compositions and is probably due to the nature of gellan gum. While high acyl gellan gum is a native polymer that forms soft and elastic gels, low acyl gellan gum is a modified polymer that forms hard and brittle gels [33]. Nevertheless, depending on the degree of swelling necessary, the choice of gellan gum type can be advantageous for specific formulations.

### 2.5. State of Bound Water

**Figure 4:**
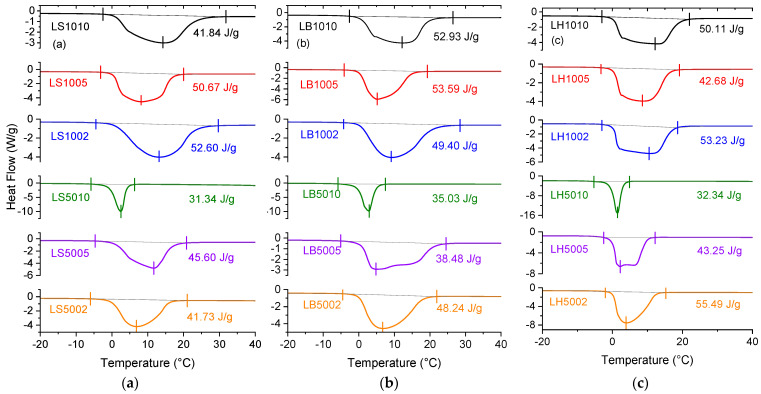
DSC thermograms and calculated Δ*H_m_* for hydrogels LS10ZZ and LS50ZZ (**a**), LB10ZZ and LB50ZZ (**b**), LH10ZZ and LH50ZZ (**c**), crosslinked with ZZ = 10, 5, and 2 wt.% of CA.

The analyses of the bound water in the hydrogels composed of 100% GGLA and 50% GGLA- 50% ST, GGLA-ST-BET, GGLA-ST-HAL, with 2, 5, and 10 wt.% of CA are presented in Figure 4.

The enthalpy of fusion for the hydrogels is calculated from the DSC thermograms presented in Figure 4, and the values of the Δ*H_m_* were used to calculate the types of water by means of Equations (2) and (3). The values of the swelling degree of the samples (S), measured fusion enthalpy (Δ*H_m_*_)_, freezable (*w_f_*), and non-freezable water (*w_nf_*) are presented in Table 1.

When the dry hydrogel meets water, the water molecules are absorbed and H-bond with the polar parts of the polymer structure, leading to water–polymer bonding [48]. As a result, the polymer network expands and exposes its most hydrophobic segments to the water, leading to the next polymer chain–water interaction. [49]. There is also the third phenomenon of osmotic diffusion. The hydrogels´ covalent and structural forces and crosslinks that keep their elastic network structure act against the hydration phenomenon. Thus, the absorption forces and water retention are counterbalanced by the polymer structure expansion till the equilibrium, i.e., the maximum water absorption [49,50]. 

From the values shown in Table 1, it is possible to observe the relation between the CA content in the hydrogels, swelling degree, and freezable and non-freezable water. The samples crosslinked with less CA have shown a high swelling capacity, which points to low polymer–polymer and high polymer–solvent (water) interactions that occur via hydrogen bonds [8]. A higher CA content promotes more crosslinking points, which leads to a lower swelling degree and stronger interaction with bound water, i.e., higher non-freezable water values. Then, more non-crosslinked hydroxyl groups in the hydrogel can lead to a higher interaction with water, therefore, a higher freezable water content [51]. 

## 3. Materials and Methods

### 3.1. Materials

For the synthesis of hydrogels, we used a commercial starch (ST, Maizena brand), low acyl gellan gum (GGLA; Kelcogel from CP Kelco, Atlanta, GA, USA), citric acid (CA) monohydrate (≥99.0% from Sigma-Aldrich, Lisboa, Portugal), halloysite (HAL; 30–70 nm × 1–3 μm nanotubes from Sigma-Aldrich), and bentonite (BET; hydrophilic powder made of ≤25 μm particles from Sigma-Aldrich). All substances were used as received.

### 3.2. Hydrogel Synthesis

We synthesized the hydrogels following Reddy and Yang’s [29] and Alimi and Workneh’s [52] adapted procedures. To obtain the nanocomposites, we used the solution intercalation method by mixing solutions of BET and/or HAL and polymer.

**Figure 5:**
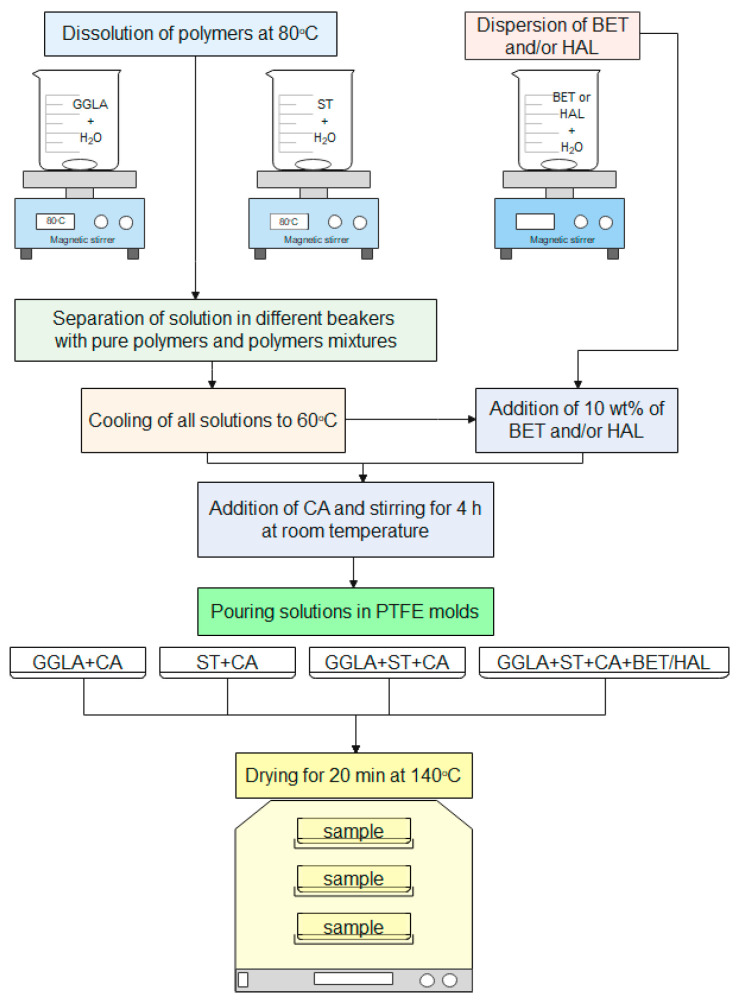
Sample preparation flow chart.

First, we dissolved 1.0 g/L of ST and, separately, 1.0 g/L of GGLA in 100 mL of ultrapure water with a resistivity superior to 18 MΩ/cm (Milli-Q, Millipore Corporation, Burlington, MA, USA) under continuous magnetic stirring at 80 °C, until total dissolution. Afterwards, a little of each solution was taken to obtain the reference samples of 100% ST and 100% GGLA. Then, we made the blends of GGLA-ST by combining their solutions in 75, 50, and 25 wt.% of GGLA. Next, the samples were chilled to 60 °C, and 2, 5, and 10 wt.% of CA were added to the GGLA, ST, and blended solutions. For the samples with BET and/or HAL, first, we added, under stirring, 10 wt.% of previously dispersed BET and/or HAL in Milli-Q water and then 2, 5, and 10 wt.% of CA. Next, all the samples were left for ~4 h, being stirred at room temperature. After that, they were dispersed into poly(tetrafluoroethylene) (PTFE) containers and left to dry at room temperature. Crosslinking was achieved by leaving these dried samples in an oven for 20 min at 140 °C. Figure 5 shows the flow chart of sample preparation. We named our samples as shown in Table 2. The reference sample, i.e., BET- and/or HAL- and starch-free hydrogels of 100 wt.% GGLA crosslinked with 10 wt.% CA was LS1010, and the hydrogel of 100 wt.% ST crosslinked with 10 wt.% CA and loaded with BET was LB0010. The general samples’ compositions are depicted in Table 3.

### 3.3. Nanocomposite Hydrogels Characterization

#### 3.3.1. Infrared Spectroscopy

To characterize hydrogels, we used attenuated total reflectance Fourier-transform spectroscopy (ATR-FTIR). We collected spectra in an IRAffinity 1 s Fourier-transform infrared spectrophotometer (Shimadzu, IZASA Scientific, Madrid, Spain), equipped with a diamond crystal. A small part of each sample was put onto a diamond crystal and pressed. The spectra were recorded in 4000–400 cm^−1^ range by averaging 64 scans, a resolution of 2 cm^−1^, at room temperature, and using the software LabSolutions IR (https://www.shimadzu.com/an/products/molecular-spectroscopy/ftir/ftir-spectroscopy-software/labsolutions-ir/index.html, accessed on 12 December 2023). The data were smoothed using FFT filter method of OriginPro 2021 software. 

#### 3.3.2. Thermal Analyses

We collected differential scanning calorimetry (DSC) data with a Mettler Toledo DSC821e Differential Scanning Calorimeter. We put small amounts of hydrogels in the aluminum pans, which were closed with perforated lids. To remove any thermal history and residual bound water we subjected the samples to a pre-treatment by heating them from 25 to 110 °C at 20 °C/min and then leaving them for 20 min at 110 °C. Then, we cooled them to −60 °C. After they reached −60 °C, we started heating the aluminum pans with our hydrogels to 400 °C at 10 °C/min under argon flow and collected the data.

#### 3.3.3. Swelling Degree

To evaluate hydrogel’s swelling degree (S), we submerged about 0.1 g of dry hydrogel in water at 25 °C. After 20 min, we removed the hydrogel from the water, surface-dried with a paper towel, and weighed in an analytical balance with ±0.00001 g precision. Next, we returned it to the water and repeated the same procedure after another 20 min. The third weight collection was taken after 1 h in water and then repeated every hour up to 8 h. After that, the data were collected after 24 and 48 h of the sample´s submersion in water. We calculated the swelling degree using Equation (1).

(1) $$S=\frac{W_{wet}-W_{dry}}{W_{dry}},$$

where *w_wet_* and *w_dry_* are the weights of hydrated and dry hydrogel, respectively [9,53].

#### 3.3.4. State of Bound Water

In the presence of excess water, the polymer is totally swollen. This water is bound to the polymer and can cause physical and chemical changes. Water can plasticize the polymer or form stable hydrogen bonds, which generate an anti-plasticizing effect. The behavior of water may change depending on the water interaction intensity with the polymer. The strongest interactions alter the characteristics of this water-bound polymer, which can be classified as freezable, bound, or freezing bound.

Freezable water (*w_f_*) presents the melting or crystallization temperature, and its enthalpy is like that of free water. In contrast, bound or non-freezable (*w_nf_*) water has no calorimetric transitions due to its stronger bond with the polymeric chain. When water is weaker associated with the polymer, it has smaller melting/crystallizing peaks and is called freezing bound water (w_fb_). The sum of these three types of water is the total water of the system [54,55,56].

Dry hydrogels were hydrated for about one hour in ultrapure water then sealed in a closed aluminum pan. The thermogram was obtained with Mettler Toledo DSC821e Differential Scanning Calorimeter by cooling the sample at 10 °C/min from ambient temperature to −60 °C, and then heating at 10 °C/min from −60 to 80 °C.

The freezable water in the samples was calculated by the integration of the endothermic melting of samples and assuming freezing water (*w_f_*) and freezing bound water (w_fb_) have the same enthalpy of Δ*H*_0_ = 334 J/g, as described for water in the literature. The value of freezable water was obtained from Equation (2).

(2) $$w_{f}=\frac{{∆H}_{m}}{{∆H}_{0}},$$

where Δ*H_m_* is the melting enthalpy obtained from integrating the peak of samples’ DSC thermogram for freezable water in the hydrogel membrane, and can be associated with freezable water; Δ*H*_0_ is the pure water melting enthalpy. The non-freezable water (*w_nf_*) amount can be calculated from Equation (3).

(3) $$w_{nf}=w_{t}-w_{f},$$

where *w_t_* is total water and *w_f_* is freezable water calculated from Equation (2) [43].

## 4. Conclusions

This paper presented the results of the synthesis and characterization of hydrogels made by crosslinking starch with low acyl gellan gum with citric acid and loaded with bentonite and/or halloysite. These hydrogels with compositions of 100, 75, 25, and 0 wt.% of GGLA to ST were crosslinked with 10, 5, and 2 wt.% of CA. The FTIR analyses revealed the characteristic peaks of organic compounds and also the evidence of crosslinking reactions between the COOH of citric acid and OH of starch and gellan gum with peaks at around 1600 cm^−1^. The DSC results have shown the degradation peaks at about 250 °C, which is typical for polysaccharides. The swelling in water properties changed depending on the samples, and the best samples were those composed of GGLA and 2 wt.% of CA and these values decreased with the addition of BET and/or HAL. The maximum absorption of 77 was for the sample with 100% of GGLA crosslinked with 2 wt.% CA (LS1002). After the addition of 10% bentonite (LB1002) or 10% halloysite (LH1002) the absorption decreased to 67 and 62, respectively. The samples’ state of bound water revealed similarities between all the compositions, especially of the samples of GGLA without and with halloysite, both crosslinked with CA. From the obtained results, it is clearly seen that the addition of starch to the GGLA hydrogels decreased their water swelling properties. However, all the presented results have shown a good compatibility between the two biopolymers, and the hydrogels have good properties that can be explored as soil conditioners in agriculture and provide support for their use as controlled-release fertilizers. Therefore, the GGLA-ST hydrogels are promising candidates for application in crop cultures.

## Figures and Tables

**Table 1:** Swelling degree (S), fusion enthalpy (Δ*H_m_*), freezable (*w_f_*), and non-freezable water (*w_nf_*) for hydrogels LS10ZZ, LS50ZZ, LB10ZZ, LB50ZZ, LH10ZZ, and LH50ZZ, crosslinked with ZZ = 10, 5, and 2 wt.% of CA.

Sample	S	Δ*H_m_* (J/g)	*w_f_* (g/g)	*w_nf_* (g/g)
LS1010	28	41.84	0.12	0.88
LS1005	31	50.67	0.15	0.85
LS1002	32	52.60	0.16	0.84
LS5010	4	31.34	0.09	0.91
LS5005	10	45.60	0.14	0.86
LS5002	18	41.73	0.12	0.84
LB1010	14	52.93	0.16	0.84
LB1005	17	53.59	0.16	0.84
LB1002	22	49.40	0.15	0.85
LB5010	3	35.03	0.10	0.90
LB5005	16	38.48	0.11	0.89
LB5002	18	48.24	0.14	0.86
LH1010	28	50.11	0.15	0.85
LH1005	22	42.68	0.13	0.87
LH1002	29	53.23	0.16	0.84
LH5010	3	32.34	0.10	0.90
LH5005	6	43.25	0.13	0.87
LH5002	21	55.49	0.17	0.83

**Table 2:** Nomenclature key for GGLA-ST and GGLA-ST-BET and/or HAL hydrogels.

Sample Name: LXYYZZ	
X	S—no BET and/or HAL, B—bentonite, and H—halloysite
YY	Gellan gum (%): 00 = 0%, 10 = 100%, 75 = 75%, 50 = 50%, and 25 = 25%
ZZ	CA (%): 02 = 2%, 05 = 5%, and 10 = 10%

**Table 3:** GGLA-ST hydrogel (H) composition, where X is S = no BET and/or HAL, with B = Bentonite or H = Halloysite. BET or HAL content in samples is 10 wt.%.

Sample Name	GGLA (wt.%)	ST (wt.%)	CA (wt.%)
LX1010	100	0	10
LX1005	100	0	5
LX1002	100	0	2
LX7510	75	25	10
LX7505	75	25	5
LX7502	75	25	2
LX5010	50	50	10
LX5005	50	50	5
LX5002	50	50	2
LX2510	25	75	10
LX2505	25	75	5
LX2502	25	75	2
LX0010	0	100	10
LX0005	0	100	5
LX0002	0	100	2

## Data Availability

Data are contained within the article.
